# Supplementary material for: Comprehensive Evaluation of Ten Actinidia arguta Wines Based on Color, Organic Acids, Volatile Compounds, and Quantitative Descriptive Analysis
Source: Foods. 2023 Sep 6;12(18):3345. doi: 10.3390/foods12183345 (PMC10529418; doi:10.3390/foods12183345)
Supplement: Supplementary file 1 [file foods-12-03345-s001.zip › foods-2563250-supplementary.pdf]

Supplementary file

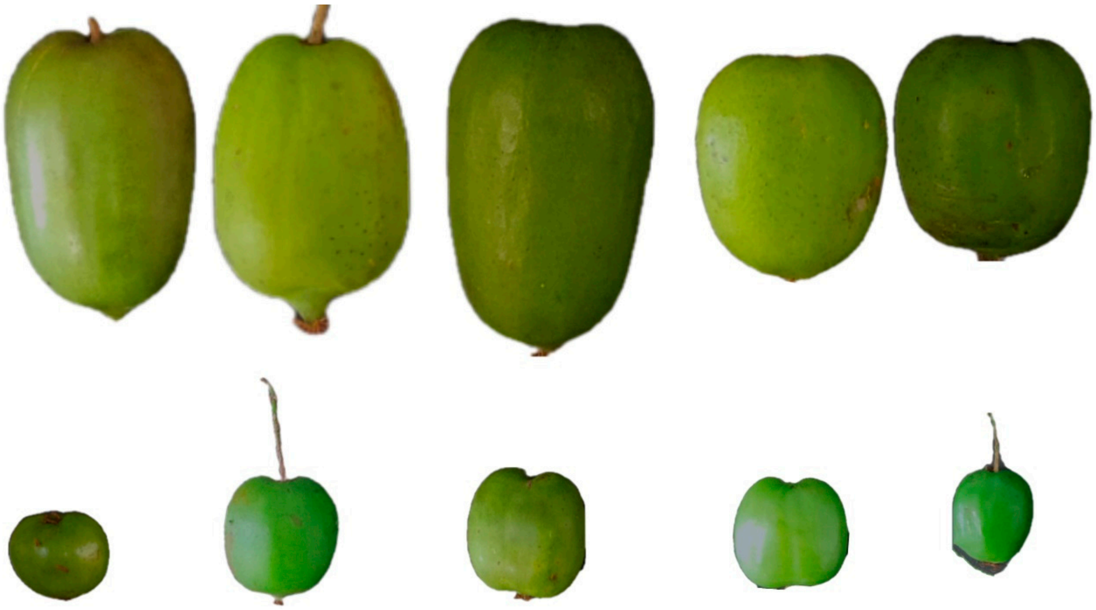

Figure S1. The picture of ten *Actinidia arguta* fruits. The first row, from left to right, they are 'Kuilv', 'Jialv', 'Longcheng No 2', 'Xinlv' and 'Wanlv'. The second row, from left to right, they are 'Fenglv', 'Pinglv', 'Tianxinbao', and 'Cuiiyu'.
